# Is quality of life related to high autistic traits, high ADHD traits and their Interaction? Evidence from a Young-Adult Community-Based twin sample

**DOI:** 10.1007/s10803-022-05640-w

**Published:** 2022-07-08

**Authors:** Simone J. Capp, Jessica Agnew-Blais, Alex Lau-Zhu, Emma Colvert, Charlotte Tye, Ümit Aydin, Alexandra Lautarescu, Claire Ellis, Tyler Saunders, Lucy O’Brien, Angelica Ronald, Francesca Happé, Gráinne McLoughlin

**Affiliations:** 1 Social Genetic and Developmental Psychiatry Centre, Institute of Psychiatry, Psychology and Neuroscience, King’s College London, London, UK; 2 Department of Psychology, School of School of Biological and Chemical Sciences, Queen Mary University London, London, UK; 3 Department of Child and Adolescent Psychiatry, Institute of Psychiatry, Psychology and Neuroscience, King’s College London, London, UK; 4 Centre for Brain and Cognitive Development, Department of Psychological Sciences, University of London, Birkbeck, London, UK; 5 Medical Sciences Division, Oxford Institute for Clinical Psychology Training and Research, University of Oxford, Oxford, UK; 6 Department of Psychology, Institute of Psychiatry, Psychology and Neuroscience, King’s College London, London, UK; 7 Centre for the Developing Brain, School of Biomedical Engineering and Imaging Sciences, King’s College London, London, UK; 8 Department of Forensic and Neurodevelopmental Sciences, Institute of Psychiatry, Psychology and Neuroscience, King’s College London, London, UK; 9 University of Edinburgh, Edinburgh, UK

**Keywords:** Autism, ADHD (attention deficit-hyperactivity disorder), Quality of life, Mental Health, Adults

## Abstract

This study explored whether high autistic traits, high attention deficit hyperactivity disorder (ADHD) traits and their interaction were associated with quality of life (QoL) in a sample of 556 of young-adult twins (Mean age 22 years 5 months, 52% Female). Four participant groups were created: high autistic traits, high ADHD traits, high autistic/ADHD traits, and low ADHD/autistic traits. High autistic traits were associated with lower QoL across domains (physical, psychological, social, and environmental). High ADHD traits associated with lower physical, psychological, and environmental QoL. The interaction of autistic and ADHD traits was not significant in any domain. While mental health difficulties were associated with lower QoL, after accounting for mental health, most relationships between autistic traits, ADHD traits and QoL remained.

Multiple research studies find that adults with neurodevelopmental conditions, such as autism spectrum conditions (henceforth autism) and attention deficit hyperactivity disorder (ADHD), experience lower quality of life (QoL) than their typically developing (TD) peers. Autism is characterised by difficulties in social communication and interaction, and restricted and repetitive behaviours and interests, whereas ADHD is identified by developmentally uncharacteristic levels of inattention, hyperactivity and impulsivity (American Psychiatric Association, 2013).

QoL is defined by the World Health Organisation (WHO) as an " individuals’ perception of their position in life in the context of the culture and value systems in which they live and in relation to their goals, expectations, standards and concerns” (p.1405; WHO, 1995). QoL is considered subjective and multidimensional, and is therefore often measured using self-report questionnaires exploring a range of life domains. Commonly used measures of QoL from the WHO (e.g., World Health Organisation Quality of Life-Bref; WHOQOL Group 1998; WHO, 1996) represent QoL in four different subdomains: Physical, Psychological, Social, Environmental QoL. However, many other measures exist and have been used in research which explore different subdomains of QoL.

Two recent systematic reviews which examined a total of 23 unique studies concluded that the majority of research has found that autistic adults reported lower levels of QoL compared with TD groups or published norms using a number of measures (Ayres et al., 2018; Sáez-Suanes & Álvarez-Couto, 2021). Although group differences are apparent between autistic and TD adults, not all autistic adults report diminished QoL (Moss et al., 2017; Oakley et al., 2021). Multiple studies have identified that higher levels of autistic traits are related to poorer QoL in several domains (Khanna et al., 2014; Knüppel et al., 2018; Lawson et al., 2020; Mason et al., 2018; van Heijst & Geurts, 2015), with some exceptions (Khanna et al., 2014; Renty & Roeyers, 2006; van Heijst & Geurts, 2015). Thus, reduced QoL associated with autism and autistic traits is commonly, but not universally, reported. Because of this, it is important to identify what factors might be related to poorer (or better) QoL for autistic people, in order to highlight key areas for support and identify those who might benefit the most from support in particular areas.

There has also been similar research exploring the QoL of adults with ADHD, although the number of studies in the area is still limited. Similar to findings from autism research, adults with ADHD have also been shown to experience lower QoL than TD groups (Chao et al., 2008; Grenwald-Mayes, 2002; Quintero et al., 2019; Thorell et al., 2019). Furthermore, increasing ADHD traits have been associated with lower QoL in adults with ADHD (Rimmerman et al., 2007; Stern et al., 2017).

ADHD is a commonly reported co-occurring condition in autistic adults. A recent meta-analysis of 63 studies estimated high current (38.5%) and lifetime (40.2%) prevalence of ADHD in autistic populations (Rong et al., 2021). Additionally, research has demonstrated high levels of phenotypic and genetic overlap among autistic and ADHD traits in both childhood and adolescence (Ronald et al., 2014) and adulthood (Riglin et al., 2021).

Despite this overlap, and findings that QoL may be lower for autistic adults and adults with ADHD, there are only three studies that have explored adult QoL in relation to autism and ADHD together, and among these, results have varied. Vincent et al., (2020) reported on a small sample of 24 French adults with a childhood diagnosis of Asperger’s syndrome, and examined self-reported QoL using the WHOQOL-BREF (WHO, 1996). They found no significant differences across all QoL domains between Asperger’s syndrome subgroups with (N = 4) and without (N = 20) ADHD. However small sample sizes limit the conclusions that may be drawn from this comparison. In contrast, a recent large-scale study of 628 autistic adults found that ADHD traits were significantly related to lower QoL in each domain, after accounting for the impact of demographic factors and autistic traits (Yerys et al., 2021). The third study examined factors related to QoL in both autistic (N = 106) and non-autistic (N = 86) adults from the Longitudinal European Autism Project (Oakley et al., 2021). Using the WHOQOL-BREF in adults, results from bivariate correlations suggested no association between ADHD traits and self-reported QoL in the psychological, social and environmental domains, but a small-moderate negative association was observed between ADHD traits and physical QoL (*r*=-.27, *p* = .04). This was the case across the combined sample (i.e. autistic and non-autistic adults) and was not reported separately by autism status. Due to insufficient complete data, they did not perform additional analyses simultaneously exploring the role of autistic and ADHD traits in adult QoL. Aside from the limited adult literature, there are several notable studies which have explored QoL in relation to autism and ADHD co-occurrence in children and adolescents. These studies have identified that children and adolescents with an autism and ADHD diagnosis had lower parent-reported QoL compared to those with autism only (Sikora et al., 2012) or ADHD only (Thomas et al., 2015). Furthermore, in a study of 6- to 10-year-old Australian children, higher autistic traits were associated with poorer quality of life in those with ADHD (Green et al., 2016).

In addition to the possible influence of autistic and ADHD traits, other factors are important when considering QoL, notably the impact of co-occurring mental health difficulties. Autistic adults (Hollocks et al., 2019; Lai et al., 2019; Lugo-Marin et al., 2019), and adults with ADHD (Chen et al., 2018; Fayyad et al., 2007), have been shown to experience higher rates of most mental health conditions than non-autistic adults/those without ADHD. Associations between autistic traits, anxiety and depression have also been found in non-autistic samples (Kanne et al., 2009; Stice & Lavner, 2019) and ADHD trait levels in adults without clinical ADHD diagnoses have been linked to the experience of anxiety and depression (Naya et al., 2021). Furthermore, there is emerging evidence that those who are autistic and have ADHD might be even more likely to experience certain mental health difficulties compared to those who are autistic or have ADHD only (Chen et al., 2015; Gordon-Lipkin et al., 2018; Solberg et al., 2019). For example, Solberg et al., (2019), using data from Norwegian population-based registries, found higher rates of anxiety in adults who were autistic with additional ADHD, compared to those with either autism or ADHD alone.

We know that mental health is an important factor to consider in relation to QoL. Previous research has identified that autistic adults (Hong et al., 2016; Knüppel et al., 2018; Lawson et al., 2020; Lin & Huang, 2019; Mason et al., 2018; Oakley et al., 2021; Vincent et al., 2020) and adults with ADHD (Quintero et al., 2019) with additional mental health difficulties or elevated mental health symptom levels report lower QoL. Despite this, no previous research has examined autism, ADHD (or their traits), and mental health together in relation to adult QoL.

The current study aims to expand upon the previous research examining QoL for those with autistic and ADHD traits. First, we aim to explore whether high autistic traits, high ADHD traits, and their interaction are associated with self-reported physical, psychological, social, and environmental QoL in a population-based sample of UK young adults. We predict that both high autistic and high ADHD traits will be negatively related to QoL across domains. Second, we aim to explore the role of additional MH difficulties in the relationship between autistic/ADHD traits and QoL. We predict that additional mental health difficulties will be significantly associated with lower QoL but that high autistic/ADHD traits will be associated with QoL after accounting for background demographic factors and mental health problems.

## Methods

### Participants

The Individual Differences in EEG in young Adults Study (IDEAS) collected data from February 2017 to May 2019. In total, 556 participants took part, with ages between 20 and 25 years (*M* = 22 years 5 months, *SD* = 12 months) and 52% were female. Both twins from 273 pairs (118 monozygotic, 154 dizygotic, 1 unknown zygosity) participated in the study, the remaining ten participants took part without their co-twin (1 from a monozygotic pair, 9 from dizygotic pairs).

IDEAS participants were all part of the Twins Early Development Study (TEDS), a community sample of over 16,000 twin pairs born in England and Wales between 1994 and 1996 (Haworth, Davis, & Plomin, 2013, Rimfeld et al., 2019). IDEAS participants were recruited by one of four routes based on previous TEDS/sub-study participation. This recruitment strategy meant the sample was enriched for those with high levels of autistic and/or ADHD traits in childhood or adolescence (see Supporting Information S1 for additional recruitment information). All participants contacted and recruited by the IDEAS study had an estimated IQ of 70 or above in adolescence. The IDEAS study required a high overall time commitment from potential participants and included several hours of questionnaires, interviews, cognitive/behavioural assessments, and computer tasks with EEG recordings. Due to the general burden of the study protocol and complexity of some tasks it was thought that those with an IQ < 70 would not be able to complete all elements of the study. This exclusion was based on adolescent data as this was the most recent timepoint with relevant data for all participants of interest.

The selective recruitment strategy employed in this study, especially with regard to adolescent autistic and ADHD traits and estimated IQ, meant that while IDEAS was community-based (rather than a clinical or convenience sample), it was not intended to be a whole community or community-representative sample.

### Procedure

IDEAS received full ethical approval from King’s College London Psychiatry, Nursing and Midwifery Research Ethics Subcommittee (RESCMR-16/17-2673).

Potential participants were contacted by the research team by telephone, email, and/or text. Interested participants were sent an information sheet and asked to book a testing session. Prior to the testing session, participants were sent a link to an online survey comprising several standardised questionnaires. This took approximately an hour to complete and was hosted using Qualtrics survey software (https://www.qualtrics.com/).

Testing sessions were carried out at the research centre or in participants’ homes. Written consent was obtained from all participants. Two researchers carried out testing sessions to allow twins to participate simultaneously if desired. During the testing session, participants completed interviews, behavioural assessments and computer-based tasks with concurrent EEG recording of electrical brain activity. Each measure was administered and scored by a trained researcher. Assessments were video recorded to allow researchers to resolve coding/scoring queries later. Testing sessions lasted 2.5–4.5 h and participants were reimbursed for travel expenses and given a £30 voucher as compensation.

## Procedure for participants also in Social Relationships (SR) Study Phase 3

A number of IDEAS participants (N = 85) had also taken part in the related SR Study Phase 3. Usually, this was several months prior to their participation in IDEAS, however some participants completed the IDEAS testing session before taking part in SR Study Phase 3. These studies employed several overlapping measures in their online questionnaire battery and in person assessments. These included the Social Responsiveness Scale-2), the World Health Organisation Quality of Life-Bref online questionnaires and the Autism Diagnostic Observation Schedule-2 which was completed in person.

To reduce time burden and practice effects, participants completed a reduced battery in the second of the two studies in which they participated. For these participants, online questionnaire measures were all completed within 1 year 9 months of the main IDEAS testing session (Med = 1 month before; Range = 1 year 9 months before- 2 months after). ADOS-2 assessments were all completed within 1 year 11 months of the main IDEAS testing session (Med = 4 months before; Range = 1 year 10 months before- 1 year 11 months after).

### Measures

Data from all measures were entered into SPSS and were cleaned and corrected for errors prior to analyses (process described in Supporting Information S2). All questionnaire measures were completed in online format, interviews and observation were carried out in person as standard.

***World Health Organisation Quality of Life-Bref*** (WHOQOL-BREF).

The WHOQOL-BREF is a commonly used measure of QoL and consists of 26 questions asking the participant to reflect on their life in the last two weeks (WHOQOL Group, 1998; WHO, 1996). Each item has a 5-point Likert response scale which varies depending on question phrasing (e.g. “How satisfied are you with your sleep?”, “How often do you have negative feelings, such as blue mood, despair, anxiety, depression?”, “How much do you enjoy life?”).

The measure consists of two summary items and 24 items divided between four subdomains (Physical, Psychological, Social, Environmental). The Physical subdomain (7 items) includes questions on physical pain, sleep, and energy for everyday activities. The Psychological domain (6 items) includes positive and negative feelings, and personal beliefs. The Social domain (3 items) covers personal relationships, social support and sex life. Finally, the Environmental domain (8 items) includes financial resources, home environment, and opportunities for leisure activities. For each domain possible scores range from 4 to 20 with higher scores indicating higher QoL (WHO, 1996).

## Social Responsiveness Scale-2 (SRS-2)

The SRS-2 adult self-report has 65 items that assess traits of autism (e.g. “I am awkward in turn taking interactions with others-for example, I have a hard time keeping up with the give-and-take of a conversation”; Constantino & Gruber 2012). Each item is rated using a 4-point Likert scale (not true = 0, sometimes true = 1, often true = 2, almost always true = 3). Raw sum scores are calculated from the numeric scores assigned to each item and range from 0 to 195. Raw scores of 68 (corresponding to T-score of 60) or higher have been used to identify participants with mild (or greater) autistic social difficulties (Constantino & Gruber, 2012).

## Barkley Adult ADHD rating scale- IV (BAARS-IV)

The BAARS-IV (Barkley, 2011) self-report version provides measure of current traits associated with ADHD in adulthood (additional questions assess age of symptom onset and impairment of functioning). For 27 item statements, participants rate how often each would have described them over the last 6 months using a 4-point Likert scale (1 = Never or rarely, 2 = Sometimes, 3 = Often, 4 = Very Often). This study used three subdomains from this measure: inattention (9 items e.g. “[I] Have difficulty organizing tasks and activities”), hyperactivity (5 items e.g. “[I] Shift around excessively or feel restless or hemmed in”), and impulsivity (4 items e.g. “[I] Have difficulty awaiting my turn”).

Items from each domain are summed to produce domain scores. An ADHD total sum score is produced from all items in the inattention, hyperactivity, and impulsivity domains. ADHD sum scores can range from 18 to 72, with higher scores related to increasing levels of ADHD like traits. Raw scores of 39 or above exceed the 93rd percentile in a general population sample of those aged 18–39 years, and represent scores of those with mild to marked ADHD traits (Barkley, 2011).

## Cognitive ability

Online versions of the Raven’s Standard Progressive Matrices (Raven, 1960) and the Mill Hill Vocabulary Scale (Raven, 1962) were used to create a general measure of cognitive ability in IDEAS. More details on these measures and items are presented in Supporting Material S3. Raven’s and Mill Hill scores were standardised and combined to create a general cognitive ability composite score. Online adaptations of these measures and ‘g score’ composites have been previously used within TEDS (Haworth et al., 2007; von Stumm & Plomin, 2015).

## Autism Diagnostic Observation schedule 2 (ADOS-2

The ADOS-2 is a gold standard semi-structured observational assessment to assess autism spectrum behavioural criteria (Lord et al., 2012). All of the participants were assessed using module 4 of the ADOS-2 (for adolescents and adults with fluent speech) which includes a range of tasks including a puzzle, creating a story with everyday items, and interview style questions. Researchers rated observed behaviours in relation to 32 autism-associated characteristics (e.g. echolalia, unusual eye contact). For each characteristic, scores range from 0 (minimal or no observed autistic-like behaviour) to 2 or 3 (marked or definite autistic-like behaviour).

A second ADOS-2 trained researcher generated their own independent scores for 62 (11.33%) ADOS-2 assessments from IDEAS participants. Percentage agreement was good on average (*M* = 88.02%, *SD* = 9.53%). This type of reliability checking is considered best practice when conducting ADOS-2 assessments.

Calibrated Severity Scores (CSS) from the ADOS-2 assessments were calculated based on updated guidelines from Hus & Lord (2014). CSS range from 1 to 10, where scores of 4 or more map on to a behavioural presentation consistent with an autism spectrum diagnosis. These CSS are used in the current study.

## Diagnostic interview for ADHD in adults 2.0 (DIVA)

The DIVA 2.0 (Kooij, 2010) is a semi-structured diagnostic interview assessing ADHD in adults, which has high diagnostic accuracy compared to clinical interview (Pettersson et al., 2018) and alternative instruments (Ramos-Quiroga et al., 2019). The interview probes participants’ experience of ADHD traits currently in adulthood and historically in childhood. A total of 18 items are assessed including nine on Inattention (e.g., “Do you often find it difficult to sustain your attention on tasks?”) and nine on Hyperactivity/Impulsivity (e.g. “Do you often feel restless?”, “Do you often find it difficult to await your turn?”). Participants are then asked whether endorsed items cause them difficulties in particular areas of their life (work/education, relationship and/or family, social contacts, free time/hobby, self-confidence/self-image).

The DIVA 2.0 assessment can be used to determine whether a participant would be likely to meet DSM-5 diagnostic criteria for adult ADHD, based on report of 5 or more symptoms of inattention and/or hyperactivity/impulsivity, recall of childhood onset of symptoms, and that these cause problems in more than one life domain (American Psychiatric Association, 2013).

## MINI International Psychiatric interview v5.0.0 (MINI)

The MINI is a structured diagnostic interview to assesses mental health problems according to DSM-IV and ICD-10 diagnostic criteria (Sheehan et al., 1998).

IDEAS used a shortened version of the MINI with modules assessing current Major Depression, Dysthymia, Panic Disorder, Agoraphobia, Social Anxiety Disorder, Obsessive-Compulsive Disorder, and Generalised Anxiety Disorder. This subset of modules was selected to provide a relatively brief, measure of common mental health conditions and those often linked to neurodevelopmental difficulties. For each condition, participants were asked a hierarchy of questions probing for symptoms, their frequency, and duration; e.g., ’Have you worried excessively or been anxious about several things over the past 6 months?’.

The specific module is discontinued after any answers are given that indicate a participant would not meet criteria for the condition. Participants who do not meet any discontinuation rules throughout a module complete all questions relating to that mental health condition. Upon finishing a module, the interviewer applies scoring rules to determine if a participant meets diagnostic criteria. Modules are completed in sequence until the end of the interview.

## TEDS Data: parental socioeconomic status (SES)

The parental SES composite variable was based on measures of parent qualifications and employment, and maternal age at birth of first child. This information was collected by TEDS when twins were 18 months old. The 18-month parental SES composite has been used and described previously (Hanscombe et al., 2012; von Stumm & Plomin, 2015).

## Participant grouping variables: autistic traits, ADHD traits, and Mental Health

Scores from the ADOS-2, SRS-2, BAARS-IV and the DIVA 2.0 were used to create two grouping variables identifying those with high levels of autistic (Aut-Hi) and/or ADHD traits (ADHD-Hi). Participants were classed as Aut-Hi if they either had an ADOS-2 CSS of four or more (indicating a behavioural presentation consistent with an autism diagnosis) or if their SRS-2 raw score was 68 or above (indicating mild to severe autistic social difficulties). This grouping was chosen to capture those with generally high levels of autistic traits or observable behaviours consistent with an autism diagnosis. Participants falling below both of these criteria were classed as low in autistic traits (Aut-Lo).

Participants were classed as ADHD-Hi if they were likely to meet DSM-5 diagnostic criteria for adult ADHD according to self-report using the DIVA 2.0 (five or more symptoms of inattention and/or hyperactivity/impulsivity, reported impairment in two or more life areas, and reported onset of some symptoms before age 12) or if their BAARS-IV total ADHD score was 39 or above (indicating mild to marked ADHD traits and related difficulties). As with the Aut-Hi grouping, this was chosen to identify young adults with generally high levels of ADHD traits or those likely to meet adult ADHD diagnostic criteria. Participants meeting neither of these criteria were classed as low in ADHD traits (ADHD-Lo).

Data from the MINI were used to identify participants with current above threshold mental health difficulties. Participants meeting diagnostic criteria for one or more mental health conditions were classed as having current mental health difficulties, while those who did not were classed as not experiencing current mental health difficulties.

### Statistical analysis

Analyses were carried out using STATA Release 16 (StataCorp, 2019). These analyses form a part of a larger project for which analyses and aims have been pre-registered (https://osf.io/cmxu8).

To compare differences in reported QoL domains among our Aut and ADHD groups, multilevel mixed effects models with random intercepts to account for clustering in the sample (i.e., related twins) were used as these allow use of the whole sample for analyses (standard regression models would not have been appropriate given that our sample contains related individuals and therefore non-independent datapoints). Each QoL domain score was the dependent variable in its own multilevel model. In each model, Aut and ADHD grouping variables have been included as indicators, as well as interaction terms between these variables. Models also included participant sex, age, cognitive ability, and parental SES based on agreed pre-registered analysis plans. Mixed effects models were re-run including the grouping variable identifying those with and without above threshold mental health difficulties (MH + vs. MH-). These were carried out to explore any remaining relationships between autistic and ADHD traits and QoL after accounting for mental health difficulties.

Multiple testing was controlled using the false discovery rate method (Benjamini & Hochberg, 1995) across all predictors included in mixed effects models. Across all models, for 60 predictors, p-values were ranked, and critical q-values were calculated using a false discovery rate of 5%.

Missing data were handled with Multiple Imputation using Chained Equations. Between 0 and 13.1% of data for each variable were missing, all of which were imputed using this method (with 20 imputed sets). Supporting Information S4 provides a full description of missing data across variables and groups, and more detail on the imputation procedure. Analyses were carried out both with complete case data and with the multiply imputed data. Results from analyses with multiply imputed data have been reported, alongside the three discrepancies in patterns of significance with complete case analyses.

The distribution of scores for non-categorical variables (QoL domain scores, age, cognitive ability and parental SES) were visually inspected via histograms. Variables considered to be non-normally distributed on visual inspection were assessed and transformed using a multivariate Box-Cox procedure (Lindsey & Sheather, 2010; Velilla, 1993). Analyses were run with both transformed and non-transformed variables, but no differences emerged. For ease of interpretation, analyses using non-transformed variables have been reported.

Raincloud plots produced using R (R Core Team, 2020) were used for visualisation of QoL scores across groups. Raincloud plots combine split-half violins (showing the probability density of the data), boxplots (showing the median and interquartile range) and raw data points jittered for improved visibility (Allen et al., 2021).

## Results

### Participant groups

Details of number of participants across groups, with and without current mental health difficulties are displayed in Table 1. Additional descriptive results are presented in Supporting Information S5, including: autistic and ADHD trait scores across groups; overlap in our high autistic and high ADHD traits groups; percentages of participants likely to meet autism and ADHD diagnostic criteria; and rates of current mental health difficulties across groups.

**Table:** Table 1

Number of Participants Across Autistic and ADHD Traits Groups and Those With and Without Current Mental Health Difficulties						
		**Low ADHD Traits** **(ADHD-Lo)**		**High ADHD Traits** **(ADHD-Hi)**		**Total**
**Low Autistic Traits** **(Aut-Lo)**						
MH-		240		95		
MH+		56		38		
**Group Total**		**296**		**133**		**429**
**(% Female)**		**(66.54%)**		**(44.27%)**		
**High Autistic Traits** **(Aut-Hi)**						
MH-		25		30		
MH+		30		42		
**Group Total**		**55**		**72**		**127**
**(% Female)**		**(34.55%)**		**(45.83%)**		
**Total**		**351**		**205**		**556**
**Note**: Additional description of group overlap and mental health status in Supporting Material S6.						
ADHD = Attention Deficit Hyperactivity Disorder. MH + = Participants with current above threshold mental health difficulties. MH-= Participants without current above threshold mental health difficulties.						

#### High autistic traits, high ADHD traits, and Quality of Life

**Fig. 1:**
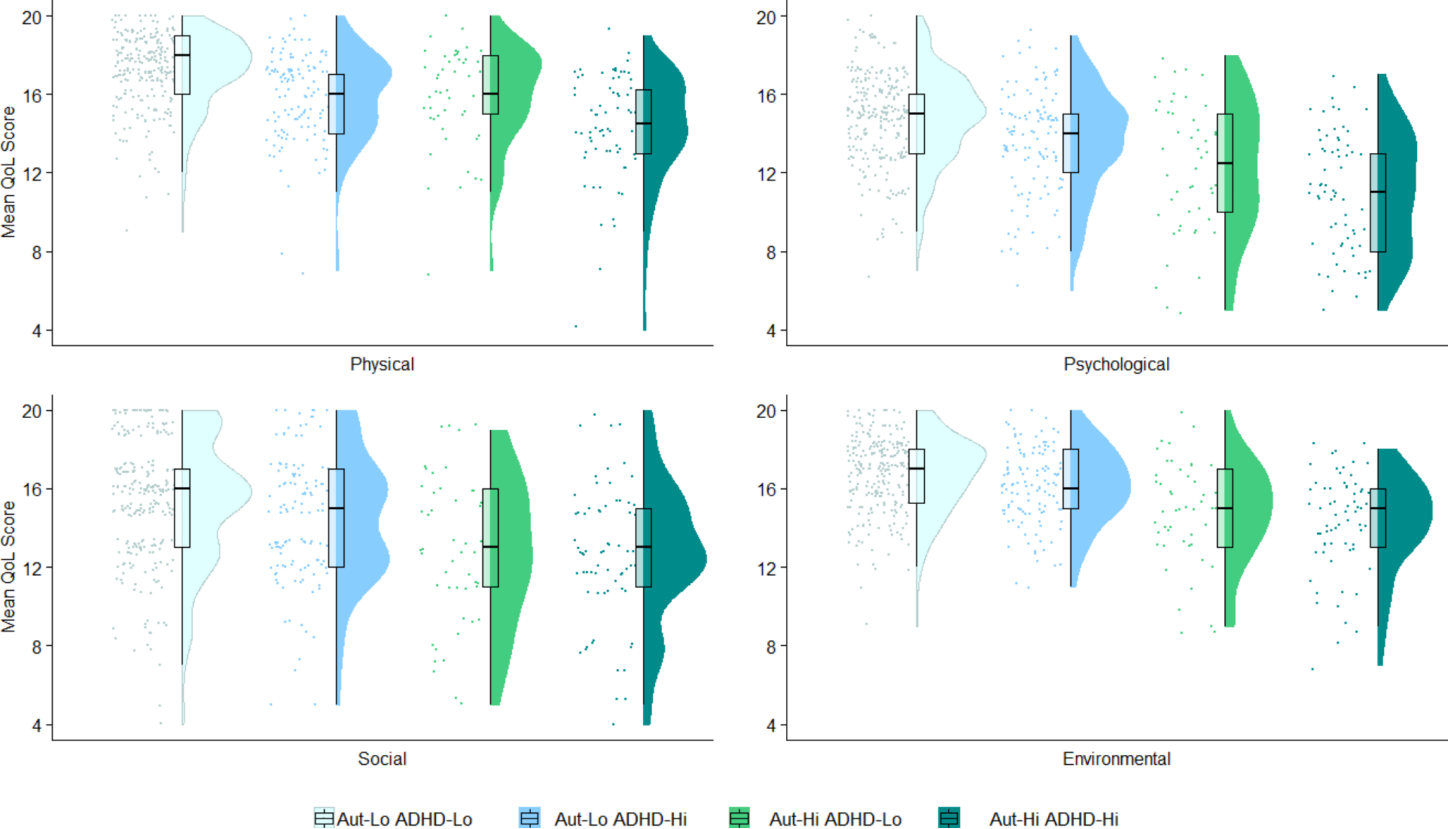
Raincloud plots showing distribution of Quality of Life (QoL) scores in participants with low autistic and ADHD traits (Aut-Lo ADHD-Lo), low autistic traits and high ADHD traits (Aut-Lo ADHD-Hi), high autistic traits and low ADHD traits (Aut-Hi ADHD-Lo), and those with high autistic and ADHD traits (Aut-Hi ADHD-Hi). For each group, jittered raw data are shown on the left, boxplots with median and interquartile range are shown in the middle, and density plots are shown on the right. See Supporting Information S6 for group means and standard deviations ADHD = Attention Deficit Hyperactivity Disorder Traits. Aut = Autistic Traits. QoL = Quality of Life

Figure 1 shows raincloud plots of the distribution of QoL domain scores across groups. Regression coefficients, p-values and corrected significance are reported in Table 2. Aut-Hi participants had significantly lower QoL in the physical, psychological, and environmental domains than the Aut-Lo group, independent of ADHD grouping. Similarly, ADHD-Hi participants had significantly lower QoL in these domains than ADHD-Lo participants, independent of Aut group. The Aut by ADHD group interaction was not significant for these domains, suggesting that also being in the ADHD-Hi group did not significantly amplify or attenuate the effect of high autistic traits on QoL, or vice versa. Finally, sex, age, parental SES, and cognitive ability were not significantly related to physical QoL scores. Cognitive ability was significantly positively associated with physical QoL (however this was only true in analyses with imputed data, not in analyses of complete data only), and males had significantly higher psychological QoL. There were no further significant relationships between sex, age, parental SES, and cognitive ability and QoL in the physical, psychological, and environmental domains.

**Table 2:** Prediction of Quality of Life from High Autistic Traits, High ADHD Traits Groups and Their Interaction

Model/ QoL Domain	Predictor	Unstandardized Coefficient	*p*-value	*q*-value	Sig. After Correction
**Physical**	**High Aut**	**-1.32**	**0.000**	**0.003**	*
	**High ADHD**	**-1.42**	**0.000**	**0.004**	*
	Aut x ADHD	0.08	0.861	0.047	-
	Age	-0.06	0.584	0.037	-
	Sex (Female)	0.35	0.111	0.025	-
	**Cognitive Ability**	**0.27**	**0.015**	**0.017**	* ^**a**^
	**Parental SES**	**-0.33**	**0.009**	**0.013**	*
**Psychological**	**High Aut**	**-2.45**	**0.000**	**0.007**	*
	**High ADHD**	**-1.19**	**0.000**	**0.008**	*
	Aut x ADHD	0.10	0.851	0.046	-
	Age	0.07	0.595	0.038	-
	**Sex (Female)**	**0.64**	**0.010**	**0.015**	*
	Cognitive Ability	0.14	0.286	0.030	-
	Parental SES	-0.09	0.533	0.035	-
**Social**	**High Aut**	**-2.31**	**0.000**	**0.010**	*
	**High ADHD**	**-0.80**	**0.037**	**0.021**	-
	Aut x ADHD	0.79	0.272	0.029	-
	Age	0.07	0.670	0.041	-
	Sex (Female)	-0.60	0.063	0.023	-
	Cognitive Ability	0.04	0.820	0.045	-
	Parental SES	0.19	0.263	0.028	-
**Environmental**	**High Aut**	**-1.65**	**0.000**	**0.001**	*
	**High ADHD**	**-0.65**	**0.003**	**0.013**	*
	Aut x ADHD	0.23	0.596	0.038	-
	Age	-0.10	0.319	0.031	-
	Sex (Female)	0.15	0.471	0.033	-
	**Cognitive Ability**	**0.21**	**0.041**	**0.022**	-
	Parental SES	0.04	0.749	0.043	-
Note: Bold denotes predictor significant at p < .05. * Predictor significant after correction for multiple comparisons ^a^ Cognitive ability met significance threshold in models with multiply imputed data, but did not meet threshold in complete case analysis ADHD = Attention Deficit Hyperactivity Disorder. Aut = Autistic Traits. Aut x ADHD = Aut and ADHD Group Interaction Term. MH = Mental Health. SES = Socioeconomic Status. Sig.=Significant. QoL = Quality of Life. q-values = Critical q-Values from Multiple Correction Procedure.					

[Figure 1]

In the social QoL domain, while those in the Aut-Hi group showed lower QoL than Aut-Lo participants, ADHD-Hi individuals did not significantly differ from those in the ADHD-Lo group after correction for multiple comparisons (although this was significant at p < .05). Sex, age, cognitive ability, and parental SES were not found to be significantly related to social QoL. Again, the Aut by ADHD group interaction was not significant.

#### Relationships with Quality of Life after Accounting for Mental Health Difficulties

Across domains, those experiencing additional mental health difficulties reported lower QoL than those without (Table 3). This was significant independent of the effects of all other variables.

**Table 3:** Prediction of Quality of Life from High Autistic Traits, High ADHD Traits Groups, and Their Interaction and Mental Health Difficulties

Model/ QoL Domain	Predictor	Unstandardized Coefficient	*p*-value	*q*-value	Sig. After Correction
**Physical**	**High Aut**	**-0.91**	**0.011**	**0.016**	*
	**High ADHD**	**-1.30**	**0.000**	**0.005**	*
	Aut x ADHD	-0.03	0.942	0.049	-
	Age	-0.06	0.563	0.036	-
	Sex (Female)	0.17	0.432	0.033	-
	**Cognitive Ability**	**0.23**	**0.032**	**0.020**	-
	**Parental SES**	**-0.29**	**0.017**	**0.018**	* ^**a**^
	**MH Difficulties**	**-1.15**	**0.000**	**0.006**	*
**Psychological**	**High Aut**	**-1.64**	**0.000**	**0.008**	*
	**High ADHD**	**-0.95**	**0.001**	**0.011**	-
	Aut x ADHD	-0.12	0.817	0.044	-
	Age	0.06	0.626	0.039	-
	Sex (Female)	0.30	0.215	0.027	-
	Cognitive Ability	0.06	0.640	0.040	-
	Parental SES	-0.01	0.915	0.048	-
	**MH Difficulties**	**-2.23**	**0.000**	**0.009**	*
**Social**	**High Aut**	**-1.92**	**0.001**	**0.012**	*
	High ADHD	-0.66	0.085	0.023	-
	Aut x ADHD	0.67	0.351	0.032	-
	Age	0.05	0.733	0.043	-
	**Sex (Female)**	**-0.78**	**0.023**	**0.018**	^**b**^
	Cognitive Ability	0.01	0.976	0.050	-
	Parental SES	0.22	0.190	0.026	-
	**MH Difficulties**	**-1.01**	**0.009**	**0.014**	*
**Environmental**	**High Aut**	**-1.19**	**0.000**	**0.002**	*
	**High ADHD**	**-0.48**	**0.028**	**0.019**	-
	Aut x ADHD	0.06	0.890	0.048	-
	Age	-0.11	0.239	0.028	-
	Sex (Female)	-0.07	0.723	0.042	-
	Cognitive Ability	0.17	0.094	0.024	-
	Parental SES	0.07	0.513	0.034	-
	**MH Difficulties**	**-1.28**	**0.000**	**0.003**	*
Note: Bold denotes predictor significant at p < .05. * Predictor significant after correction for multiple comparisons. ^a^ Parental SES met significance threshold in models with multiply imputed data, but did not meet threshold in complete case analysis ^b^ Sex met significance in complete case analysis only, but not in models with multiply imputed data ADHD = Attention Deficit Hyperactivity Disorder. Aut = Autistic Traits. Aut x ADHD = Aut and ADHD Group Interaction Term. MH = Mental Health. SES = Socioeconomic Status. Sig.=Significant. QoL = Quality of Life. q-values = Critical q-Values from Multiple Correction Procedure					

Relationships with high autistic traits and QoL remained after accounting for current mental health difficulties. Relationships between high ADHD traits and physical and psychological QoL remained significant, however the association of high ADHD traits and environmental QoL became non-significant after accounting for mental health. parental SES remained associated with physical QoL (although only in analyses with imputed data), but sex was no longer significantly related to psychological QoL. We also note that in these analyses, sex was significantly related to social QoL in complete case analyses but was not significant when using multiply imputed data.

These results suggest that poorer QoL in those with high autistic traits (and ADHD traits with respect to physical and psychological QoL) is not solely due to the experience of additional mental health difficulties. However, the poorest QoL was reported by those with high autistic traits and additional mental health difficulties.

## Discussion

This is the first study to examine subjective QoL in relation to high autistic traits, high ADHD traits, and their interaction in adulthood. Our focus on the young adult period is important because of the relative under-representation of adult samples compared to child and adolescent studies in autism research (Mason et al., 2022; Mukaetova-Ladinska et al., 2012). Our findings shed light on the experiences of young adults with high autistic and/or ADHD traits and how these interact. Furthermore, this study also found a significant association between QoL and current mental health difficulties, but that high autistic traits remained significantly associated with QoL after adjusting for these and other demographic characteristics.

### High autistic traits are Associated with Lower Quality of Life among Young adults

We found high autistic traits were associated with poorer QoL across all domains. Critically, this was independent of age, sex, cognitive ability, parental SES, and ADHD traits, suggesting that autistic traits are independently associated with QoL over and above these other factors. This is in line with studies which report group differences across multiple QoL domains between autistic and TD adults (Bishop-Fitzpatrick et al., 2018; Hamm & Yun, 2019; Jennes-Coussens et al., 2006; Lin, 2014; Lin & Huang, 2019; Mason et al., 2018; Oakley et al., 2021; van Heijst & Geurts, 2015). Our findings suggest adults with high levels of autistic traits, irrespective of whether they meet full autism diagnostic criteria, still experience lower QoL in every domain than low traits counterparts.

#### Quality of life and high ADHD traits

Participants in the high ADHD group had significantly lower physical, psychological and environmental QoL compared to those with lower ADHD traits, independent of autistic traits and demographic variables. However high ADHD traits were not independently associated with social QoL. This study adds further evidence that ADHD traits are linked to lower physical QoL in adulthood both in mixed sample (like ours and Oakley et al., 2021), and specifically for autistic adults (Yerys et al., 2021). This association could be due to the higher rates of several physical health problems (Instanes et al., 2016) and multi-morbidity (Stickley et al., 2017) in adults with ADHD. Therefore promoting good physical health and preventing health problems, where possible, among those with ADHD or high ADHD traits may be particularly important for improving their QoL. Our findings also suggested that high ADHD traits were also linked to poorer QoL in the psychological and environmental, but not social QoL domains. These results fit with some, but not all, previous findings, e.g. Yerys et al., (2021), but not Oakley et al., (2021), found that ADHD traits were associated with worse psychological, social and environmental QoL. Further research is needed to address this inconsistency in the literature and begin to explain whether discrepancies in findings to date are likely to be due to sample size differences, sample differences, measurement approaches and/or controlling for different demographic factors.

#### Quality of life and the Autistic/ADHD Traits Interaction

We also tested whether the effect of high autistic traits on QoL was quantitatively different between those with high or low ADHD traits via an interaction between the autistic and ADHD groups. Across all QoL domains we found no significant interaction effects after correction for multiple comparisons. This indicates that the negative association of high autistic traits with QoL is not significantly different among those who also have high ADHD traits (and vice versa). Our ADHD grouping had a significant independent effect in relation to physical, psychological, and environmental (but not social) QoL. Therefore, while high ADHD traits do not seem to exacerbate the effect of high autistic traits on QoL, those with high levels of both traits are still likely to experience the poorest QoL, as having high traits in each area is independently associated with lower QoL.

#### Additional impact of Mental Health Difficulties

We found that above threshold mental health difficulties were, significantly and independently, associated with lower QoL across all domains. After accounting for the impact of mental health, we still found largely the same pattern of significant results in relation to the autistic and ADHD trait groups. Specifically, those in the high autistic traits group still reported significantly lower QoL across all domains, independent of their mental health status. The independent effect of ADHD group in the physical and psychological domains also remained, although the relationship with high ADHD traits and lower environmental QoL was no longer significant. Lower environmental QoL in those with high ADHD traits may be largely due to co-occurring mental health difficulties or inequalities arising from mental health status.

In relation to the perseverance of the relationship between high autistic traits and lower QoL after accounting for mental health, our results are consistent with Mason et al., (2018) who found that continuous autistic traits and having a mental health condition were both significant independent predictors of self-reported QoL across domains in 370 autistic adults. Coversely, Oakley et al., (2021) found no effect of autistic traits in their adult sample (106 autistic, 86 non-autistic) after accounting for continuous anxiety and depressive traits. They suggested that this might be because (a) the areas of QoL most affected by core autism characteristics are not reflected in the WHOQOL-BREF or (b) that at older ages individuals develop compensatory strategies that attenuate the link between autism characteristics and QoL. Our findings, which used the WHOQOL-BREF with adults of a similar mean age, suggest that these factors do not explain the conflicting findings. In our study and Mason et al.’s (2018), mental health was represented using a single dichotomous variable (any vs. no mental health difficulty), so it may be the use of multiple continuous mental health scores that explain Oakley et al’s different results. In the current study, we chose to explore differences between those with any vs. no mental health difficulties (in line with previous work by Mason et al., 2018). It should also be noted that the reduced subset of the MINI employed in this study only examined a few different types of mental health difficulties. Therefore, further research should explore the impact of a broader range of mental health difficulties and consider how different types of co-occurring mental health difficulties are related to QoL, alongside autism, ADHD (or traits) and their overlap. Nonetheless, our findings would suggest that support aiming to improve QoL for adults with neurodevelopmental conditions or mental health difficulties should consider the role of autistic traits, ADHD traits, and mental health difficulties simultaneously.

### Relationships with Age, sex, cognitive ability, and parental SES

Compared to the more widespread associations that we identified between autistic traits, ADHD traits, mental health and QoL, we found fewer significant relationships with age, sex, cognitive ability and parental SES. Before accounting for the role of mental health, higher cognitive ability and parental SES were associated with physical QoL, sex was associated with social QoL and cognitive ability was associated with environmental QoL. After accounting for the role of mental health, parental SES and its association with physical QoL was the only one that remained significant. These relationships, or lack thereof, between QoL and some of these demographic factors should be interpreted in light of a number of considerations. Our measure of SES is based on parental information from when twins were 18 months of age. This measure was selected as a covariate in analyses because of its high coverage of the sample and use of existing records introduced no additional burden on participants, who were already completing a lengthy testing and questionnaire battery for this study. However, it should be acknowledged that this measure is likely to be far less relevant to current QoL than information based on the young adult’s current SES. Similarly, while sex at birth has been used in analyses, young adult gender identity, which may be different, may be more relevant to current QoL.

It is also important to note that there were three differences in patterns of significance between our analyses with multiply imputed data and those using complete case data only. These related to the role of cognitive ability (physical QoL model), parental SES (physical QoL model accounting for mental health), and sex (social QoL models accounting for mental health). These differences could be due to a number of factors; they may indicate that multiply imputed data better account for processes that produce missing data, or a poorly specified multiple imputation model producing invalid results compared to complete data. Since the reasons cannot be determined in the current design, these particular results should be considered with more caution.

#### Strengths and Limitations

The inclusion of a moderate sized, community-based sample of young adults (including 260 with high autistic traits, high ADHD traits or both) is a key strength of this study. Many QoL studies in relation to autism and autistic traits have focused on small and clinically-derived samples (Ayres et al., 2018; Sáez-Suanes & Álvarez-Couto, 2021). There is also relatively little research into QoL and ADHD in adults (Agarwal et al., 2012). As such this study extends the current knowledge base and forms a bridge between autism and ADHD QoL research. However, some limitations should be considered with respect to this sample and our study. Studying a sample of twins, may in itself affect our findings on QoL in young adulthood. Twins may differ from non-twin populations in relevant ways; for example, twins have greater sibling attachments (with their twin) in adult life compared to non-twin siblings (de Oliveira Landenberger et al., 2021; Fraley & Tancredy, 2012). This could be important as previous research has highlighted that social/informal support (e.g., Bishop-Fitzpatrick et al., 2018), social networks (Mason et al., 2018), and lower loneliness (Lin & Huang, 2019) are related to more positive QoL in autistic adults. For adults with ADHD, improved QoL is related to having a friend (Rimmerman et al., 2007). Thereby, differences in the social lives and supports of twins might influence their QoL (and its interrelationship with other factors) compared to non-twins. Therefore, it would be beneficial for our findings to be replicated in non-twin samples, and further research could examine the role of twin-status on QoL in adulthood.

Exclusion of individuals with low IQ (estimated from adolescent data) in this study means that our sample is not representative of individuals across the full continuum of autistic and ADHD traits in adulthood. This represents a key bias in our sample for several reasons. Adolescent estimated IQ data were used for this exclusion as this was the most recent timepoint with available data for all participants of interest (i.e. those from previous TEDS sub-studies). Typically, IQ does show a high degree of stability between adolescence and young adulthood (Deary et al., 2000) however we cannot rule out that participants who would now have an IQ in the average range may have been excluded. Since IQ may not be a reliable indicator of functional abilities in those on the autism spectrum (Alvares et al., 2020), it is also possible that some excluded participants would have proved able to complete the long and complex battery of tasks in IDEAS.

Future work building on our findings should seek to include those with a broader range of cognitive and functional abilities. Some studies of autistic adults’ QoL have used parental proxy-reports to capture the QoL of adults with intellectual disability (Hong et al., 2016; Knüppel et al., 2018; Moss et al., 2017). However, the validity of proxy-report QoL has been questioned because these ratings seem to be consistently lower than self-report (Jonsson et al., 2017). Overall, it has been highlighted that more needs to be done to include the experiences of those often excluded from studies using self-report QoL measures (e.g. Shogren et al., 2021).

The use of high autistic/ADHD traits, rather than relying on clinical diagnoses, allowed us to examine the QoL of those who have substantial autistic and/or ADHD traits without some of the possible biases associated with clinical samples. Therefore, this may improve inclusion of groups who are typically under- or mis-diagnosed and hence not fully represented in clinical settings (e.g. females; Loomes et al., 2017). Nonetheless, we highlight that the IDEAS sample is also selected, albeit differently to clinical studies, so is likely to present with its own unique biases.

Despite some of the strengths of our high traits approach it is important to consider that results from more inclusive criteria may be less representative of the experience of clinically diagnosed autistic or ADHD adults, and may fail to reflect their poor experiences in relation to QoL. However, evidence suggests that the continuum of autistic (Lundström et al., 2012; Ronald et al., 2006; Robinson et al., 2011), and ADHD (Larsson et al., 2012; Stergiakouli et al., 2015) traits are underpinned by the same genetic variation as the diagnosed conditions. From this perspective, our findings from high traits groups should still provide some valuable information when considering the QoL of those diagnosed with autism and/or ADHD.

There are several other considerations concerning the selection of high and low ADHD groups and the type of measures used. Our high ADHD traits group included those who appeared to meet full current ADHD diagnostic criteria as well as those reporting generally high levels of traits. As such, our grouping does not distinguish between those with persistent levels of ADHD/traits across the lifespan, and those who may be reporting these experiences for the first time in adulthood. Ongoing debate remains concerning to what extent adult-onset ADHD is similar to or distinct from persisting childhood-onset ADHD in terms of aetiology and neural mechanisms (Asherson & Agnew-Blais, 2019; Polanczyk et al., 2019). Further work should examine differences in QoL between ADHD groups reporting different patterns of onset/persistence, and whether they present similar interrelationships with QoL and autistic traits as we have found here.

Concerning the measures used in this study, there was a heavy emphasis on current self-report information, for autistic and ADHD traits, mental health and QoL (the ADOS-2 was the only current observational measure). In some respects, this is a strength of the study as it emphasizes the relationships between the current subjective experiences of young adults. However, these results may be questioned if young adults themselves do not provide the best information on their own traits and difficulties. For example, in relation to autism and ADHD there are concerns that we do not have strong evidence as to when/if self-reports are the most useful source of information (Hartman et al., 2016). Related to this issue, the reliance on self-report information means that we cannot rule out a response bias; those likely to endorse high autistic or ADHD traits, may also be likely to endorse poorer QoL or mental health, irrespective of how these constructs actually manifest or would be judged by an observer/clinician.

An additional consideration is the length of time between participants completing different measures within the study. Due to the length of the testing and questionnaire battery employed in IDEAS, participants often completed online questionnaire measures (WHOQOL-BREF, SRS-2, BAARS-IV, Cognitive Ability) separately to in person assessments (ADOS-2, DIVA 2.0, MINI). Furthermore, a small subset of participants completed some measures (WHOQOL-BREF, SRS-2, ADOS-2) at different time points with the related SR Study. Given that the WHOQOL-BREF asks participants to assess their QoL within the last two weeks, the gap between completion dates across measures for some participants, raises additional questions in the interpretation of our findings. Nonetheless, it is encouraging that our key findings in relation to QoL, high autistic traits, high ADHD traits and mental health seem to have been robust to temporal differences in measure completion.

Furthermore, for the current study we focused on a relatively narrow range of additional factors in relation to autistic traits, ADHD traits, their possible interaction and QoL. However, we recognize that there are likely to be many other important factors influencing the relationships we have examined. Future research is needed to address the roles of physical health, medication use, independence, and access to services in relation to QoL in autism/ADHD groups. Camouflaging of neurodivergent traits, in particular, deserves inclusion in studies of QoL in these groups, since ‘masking’ has been reported to be negatively associated with wellbeing in autism (Cook et al., 2021) and is beginning to be explored in ADHD research (Young et al., 2020).

Finally, while we have chosen to focus on current traits and experiences, future work may wish to consider longitudinal associations between childhood autistic and ADHD traits and adult QoL. These findings may be of greater utility clinically by identifying areas for early support for autistic children and those with ADHD to reduce the likelihood of poor QoL outcomes in adulthood.

## Conclusions

Our study found that young adults with high autistic traits experienced lower QoL across all domains than their low traits counterparts. Therefore, those with high levels of traits, not just diagnosed autistic adults, may benefit from targeted support to improve their QoL. Additionally, those with high autistic traits with co-occurring high levels of ADHD traits have especially poor QoL in relation to their physical and psychological health and may be in particular need of support in these areas. The types of support that would be beneficial are likely to differ based on an individual’s particular QoL profile and circumstances. Therefore, future research should specifically aim to identify which supports promote the best QoL for specific groups, taking into account their traits of both autism and ADHD, as well as wider factors.

Our findings also have implications beyond clinical service provision specific to neurodevelopmental disorders. Given the association of high autistic and ADHD traits with lower quality of life, it may be important for general mental health services to routinely screen for and consider the role of co-occurring autistic and ADHD traits in the design and implementation of interventions and treatments for general mental health needs.

## References

[CR1] Agarwal, R., Goldenberg, M., Perry, R., & IsHak, W. W. (2012). The quality of life of adults with attention deficit hyperactivity disorder: a systematic review. *Innovations in Clinical Neuroscience, 9*(5–6), 10–21. Retrieved from https://www.ncbi.nlm.nih.gov/pubmed/22808445 PMC3398685 22808445

[CR2] Allen, M., Poggiali, D., Whitaker, K., Whitaker, K., Marchall, T. R., van Langen, J., & Kievit, R. A. (2021). Raincloud plots: a multi-platform tool for robust data visualization [version 2; peer review: 2 approved]. *Wellcome Open Research*, *4*(63), 10.12688/wellcomeopenres.15191.2 10.12688/wellcomeopenres.15191.1 PMC6480976 31069261

[CR3] Alvares GA, Bebbington K, Cleary D, Evans K, Glasson EJ, Maybery MT, Whitehouse AJ (2019). The misnomer of ‘high functioning autism’: Intelligence is an imprecise predictor of functional abilities at diagnosis. Autism.

[CR4] American Psychiatric Association (2013). Diagnostic and statistical manual of mental disorders.

[CR5] Asherson P, Agnew-Blais J (2019). Annual Research Review: Does late-onset attention-deficit/hyperactivity disorder exist?. Journal of Child Psychology and Psychiatry.

[CR6] Ayres M, Parr JR, Rodgers J, Mason D, Avery L, Flynn D (2018). A systematic review of quality of life of adults on the autism spectrum. Autism.

[CR7] Barkley RA (2011). Barkley Adult ADHD Rating Scale-IV.

[CR8] Benjamini Y, Hochberg Y (1995). Controlling the False Discovery Rate: A Practical and Powerful Approach to Multiple Testing. Journal of the Royal Statistical Society: Series B (Methodological).

[CR9] Bishop-Fitzpatrick L, Mazefsky CA, Eack SM (2018). The combined impact of social support and perceived stress on quality of life in adults with autism spectrum disorder and without intellectual disability. Autism.

[CR10] Chao CY, Gau SS, Mao WC, Shyu JF, Chen YC, Yeh CB (2008). Relationship of attention-deficit-hyperactivity disorder symptoms, depressive/anxiety symptoms, and life quality in young men. Psychiatry and Clinical Neurosciences.

[CR11] Chen MH, Wei HT, Chen LC, Su TP, Bai YM, Hsu JW, Chen YS (2015). Autistic spectrum disorder, attention deficit hyperactivity disorder, and psychiatric comorbidities: A nationwide study. Research in Autism Spectrum Disorders.

[CR12] Chen Q, Hartman CA, Haavik J, Harro J, Klungsøyr K, Hegvik TA, Larsson H (2018). Common psychiatric and metabolic comorbidity of adult attention-deficit/hyperactivity disorder: A population-based cross-sectional study. PLoS One.

[CR13] Constantino JN, Gruber CP (2012). Social Responsiveness Scale–Second Edition (SRS-2).

[CR14] Cook J, Hull L, Crane L, Mandy W (2021). Camouflaging in autism: A systematic review. Clinical Psychology Review.

[CR15] Deary IJ, Whalley LJ, Lemmon H, Crawford JR, Starr JM (2000). The Stability of Individual Differences in Mental Ability from Childhood to Old Age: Follow-up of the 1932. Scottish Mental Survey Intelligence.

[CR16] de Oliveira Landenberger R, Lucci K, Frayze David T, França V, Ferreira I, de Souza Fernandes E, Otta E (2021). Hierarchy of attachment figures among adult twins and non-twins. Personality and Individual Differences.

[CR17] Fayyad J, De Graaf R, Kessler R, Alonso J, Angermeyer M, Demyttenaere K, Jin R (2007). Cross-national prevalence and correlates of adult attention-deficit hyperactivity disorder. British Journal of Psychiatry.

[CR18] Fraley RC, Tancredy CM (2012). Twin and Sibling Attachment in a Nationally Representative Sample. Personality and Social Psychology Bulletin.

[CR19] Gordon-Lipkin, E., Marvin, A. R., Law, J. K., Lipkin, P. H., & Pediatrics (2018). 141(4),e20171377. doi:10.1542/peds.2017-1377 10.1542/peds.2017-1377 29602900

[CR20] Green JL, Sciberras E, Anderson V, Efron D, Rinehart N (2016). Association between autism symptoms and functioning in children with ADHD. Archives of Disease in Childhood.

[CR21] Grenwald-Mayes G (2002). Relationship between current quality of life and family of origin dynamics for college students with Attention-Deficit/Hyperactivity Disorder. Journal Of Attention Disorders.

[CR22] Hamm J, Yun J (2019). Influence of physical activity on the health-related quality of life of young adults with and without autism spectrum disorder. Disability And Rehabilitation.

[CR23] Hanscombe KB, Trzaskowski M, Haworth CM, Davis OS, Dale PS, Plomin R (2012). Socioeconomic status (SES) and children’s intelligence (IQ): in a UK-representative sample SES moderates the environmental, not genetic, effect on IQ. PLoS One.

[CR24] Hartman CA, Geurts HM, Franke B, Buitelaar JK, Rommelse NNJ (2016). Changing ASD-ADHD symptom co-occurrence across the lifespan with adolescence as crucial time window: Illustrating the need to go beyond childhood. Neuroscience & Biobehavioral Reviews.

[CR25] Haworth CM, Harlaar N, Kovas Y, Davis OS, Oliver BR, Hayiou-Thomas ME, Plomin R (2007). Internet cognitive testing of large samples needed in genetic research. Twin Research And Human Genetics : The Official Journal Of The International Society For Twin Studies.

[CR26] Hollocks MJ, Lerh JW, Magiati I, Meiser-Stedman R, Brugha TS (2019). Anxiety and depression in adults with autism spectrum disorder: a systematic review and meta-analysis. Psychological Medicine.

[CR27] Hong J, Bishop-Fitzpatrick L, Smith LE, Greenberg JS, Mailick MR (2016). Factors Associated with Subjective Quality of Life of Adults with Autism Spectrum Disorder: Self-Report Versus Maternal Reports. Journal of Autism and Developmental Disorders.

[CR28] Hus V, Lord C (2014). The autism diagnostic observation schedule, module 4: revised algorithm and standardized severity scores. Journal Of Autism And Developmental Disorders.

[CR29] Instanes JT, Klungsøyr K, Halmøy A, Fasmer OB, Haavik J (2016). Adult ADHD and Comorbid Somatic Disease: A Systematic Literature Review. Journal of Attention Disorders.

[CR30] Jennes-Coussens M, Magill-Evans J, Koning C (2006). The quality of life of young men with Asperger syndrome: A brief report. Autism.

[CR31] Jonsson U, Alaie I, Löfgren Wilteus A, Zander E, Marschik PB, Coghill D, Bölte S (2017). Annual Research Review: Quality of life and childhood mental and behavioural disorders – a critical review of the research. Journal of Child Psychology and Psychiatry.

[CR33] Kanne SM, Christ SE, Reiersen AM (2009). Psychiatric Symptoms and Psychosocial Difficulties in Young Adults with Autistic Traits. Journal of Autism and Developmental Disorders.

[CR34] Khanna R, Jariwala-Parikh K, West-Strum D, Mahabaleshwarkar R (2014). Health-related quality of life and its determinants among adults with autism. Research in Autism Spectrum Disorders.

[CR35] Knüppel A, Telléus GK, Jakobsen H, Lauritsen MB (2018). Quality of life in adolescents and adults with autism spectrum disorder: Results from a nationwide Danish survey using self-reports and parental proxy-reports. Research in Developmental Disabilities.

[CR36] Lai MC, Kassee C, Besney R, Bonato S, Hull L, Mandy W, Ameis SH (2019). Prevalence of Co-Occurring Mental Health Diagnoses in the Autism Population: A Systematic Review and Meta-Analysis. Lancet Psychiatry.

[CR37] Larsson H, Anckarsater H, Råstam M, Chang Z, Lichtenstein P (2012). Childhood attention-deficit hyperactivity disorder as an extreme of a continuous trait: a quantitative genetic study of 8,500 twin pairs. Journal of Child Psychology and Psychiatry.

[CR38] Lawson LP, Richdale AL, Haschek A, Flower RL, Vartuli J, Arnold SRC, Trollor JN (2020). Cross-sectional and longitudinal predictors of quality of life in autistic individuals from adolescence to adulthood: The role of mental health and sleep quality. Autism.

[CR39] Lin LY (2014). Quality of life of Taiwanese adults with autism spectrum disorder. PLoS One.

[CR40] Lin LY, Huang PC (2019). Quality of life and its related factors for adults with autism spectrum disorder. Disability And Rehabilitation.

[CR41] Lindsey C, Sheather S (2010). Power Transformation via Multivariate Box–Cox. The Stata Journal: Promoting communications on statistics and Stata.

[CR42] Loomes R, Hull L, Mandy WPL (2017). What Is the Male-to-Female Ratio in Autism Spectrum Disorder? A Systematic Review and Meta-Analysis. Journal of the American Academy of Child & Adolescent Psychiatry.

[CR43] Lord C, Rutter M, DiLavore PC, Risi S, Gotham K, Bishop S (2012). Autism Diagnostic Observation Schedule (ADOS) 2.

[CR44] Lugo-Marin J, Magan-Maganto M, Rivero-Santana A, Cuellar-Pompa L, Alviani M, Jenaro-Rio C, Canal-Bedia R (2019). Prevalence of psychiatric disorders in adults with autism spectrum disorder: A systematic review and meta-analysis. Research in Autism Spectrum Disorders.

[CR45] Lundström S, Chang Z, Råstam M, Gillberg C, Larsson H, Anckarsäter H, Lichtenstein P (2012). Autism Spectrum Disorders and Autisticlike Traits: Similar Etiology in the Extreme End and the Normal Variation. Archives of General Psychiatry.

[CR46] Mason D, McConachie H, Garland D, Petrou A, Rodgers J, Parr JR (2018). Predictors of quality of life for autistic adults. Autism Research.

[CR47] Mason D, Stewart GR, Capp SJ, Happé F (2022). The Rising Tide of “Gerontautism”. Autism in Adulthood.

[CR48] Moss P, Mandy W, Howlin P (2017). Child and Adult Factors Related to Quality of Life in Adults with Autism. Journal of Autism and Developmental Disorders.

[CR49] Mukaetova-Ladinska EB, Perry E, Baron M, Povey C (2012). Ageing in people with autistic spectrum disorder. International Journal Of Geriatric Psychiatry.

[CR50] Naya N, Tusuji T, Nishigaki N, Sakai C, Chen Y, Jung S, Kosaka H (2021). The Burden of Undiagnosed Adults With Attention-Deficit/Hyperactivity Disorder Symptoms in Japan: A Cross-Sectional Study. Cureus.

[CR51] Oakley BF, Tillmann J, Ahmad J, Crawley D, San Jose Caceres A, Holt R, Loth E (2021). How do core autism traits and associated symptoms relate to quality of life? Findings from the Longitudinal European Autism Project. Autism.

[CR52] Pettersson R, Soderstrom S, Nilsson KW (2018). Diagnosing ADHD in Adults: An Examination of the Discriminative Validity of Neuropsychological Tests and Diagnostic Assessment Instruments. Journal Of Attention Disorders.

[CR53] Polanczyk GV, Casella C, Jaffee SR (2019). Commentary: ADHD lifetime trajectories and the relevance of the developmental perspective to Psychiatry: reflections on Asherson and Agnew-Blais, (2019). Journal of Child Psychology and Psychiatry.

[CR54] Quintero J, Morales I, Vera R, Zuluaga P, Fernandez A (2019). The Impact of Adult ADHD in the Quality of Life Profile. Journal Of Attention Disorders.

[CR55] R Core Team (2020). R: A language and environment for statistical computing. R Foundation for Statistical Computing, Vienna, Austria. URL http://www.R-project.org/

[CR56] Ramos-Quiroga JA, Nasillo V, Richarte V, Corrales M, Palma F, Ibanez P, Kooij JJS (2019). Criteria and Concurrent Validity of DIVA 2.0: A Semi-Structured Diagnostic Interview for Adult ADHD. Journal Of Attention Disorders.

[CR57] Raven JC (1960). Guide to the Standard Progressive Matrices.

[CR58] Raven JC (1962). Extended Guide to using the Mill Hill Vocabulary Scale with the Progressive Matrices Scales.

[CR59] Renty JO, Roeyers H (2006). Quality of life in high-functioning adults with autism spectrum disorder: The predictive value of disability and support characteristics. Autism.

[CR60] Riglin L, Leppert B, Langley K, Thapar AK, O’Donovan MC, Smith D, Thapar G (2021). Investigating attention-deficit hyperactivity disorder and autism spectrum disorder traits in the general population: What happens in adult life?. Journal Of Child Psychology And Psychiatry.

[CR61] Rimfeld K, Malanchini M, Spargo T, Spickernell G, Selzam S, McMillan A, Plomin R (2019). Twins Early Development Study: A Genetically Sensitive Investigation into Behavioral and Cognitive Development from Infancy to Emerging Adulthood. Twin Research and Human Genetics.

[CR62] Rimmerman A, Yurkevich O, Birger M, Azaiza F, Elyashar S (2007). Quality of life of Israeli adults with borderline intelligence quotient and attention-deficit/hyperactivity disorder. International Journal Of Rehabilitation Research.

[CR63] Robinson EB, Koenen KC, McCormick MC, Munir K, Hallett V, Happé F, Ronald A (2011). Evidence That Autistic Traits Show the Same Etiology in the General Population and at the Quantitative Extremes (5%, 2.5%, and 1%). Archives Of General Psychiatry.

[CR64] Ronald A, HappÉ F, Bolton P, Butcher LM, Price TS, Wheelwright S, Plomin R (2006). Genetic Heterogeneity Between the Three Components of the Autism Spectrum: A Twin Study. Journal of the American Academy of Child & Adolescent Psychiatry.

[CR65] Ronald A, Larsson H, Anckarsater H, Lichtenstein P (2014). Symptoms of autism and ADHD: a Swedish twin study examining their overlap. Journal Of Abnormal Psychology.

[CR66] Rong Y, Yang CJ, Jin Y, Wang Y (2021). Prevalence of attention-deficit/hyperactivity disorder in individuals with autism spectrum disorder: A meta-analysis. Research in Autism Spectrum Disorders.

[CR67] Sáez-Suanes GP, Álvarez-Couto M (2021). Factors Associated with Quality of Life in Adults with Autism Spectrum Disorder: A Systematic Review. Review Journal of Autism and Developmental Disorders.

[CR68] Sheehan, D. V., Lecrubier, Y., Sheehan, K. H., Amorim, P., Janavs, J., Weiller, E., & Dunbar, G. C. (1998). The Mini-International Neuropsychiatric Interview (M.I.N.I.): the development and validation of a structured diagnostic psychiatric interview for DSM-IV and ICD-10. *J Clin Psychiatry, 59 Suppl 20*, 22–33;quiz 34–57. Retrieved from https://www.ncbi.nlm.nih.gov/pubmed/9881538 9881538

[CR69] Shogren KA, Bonardi A, Cobranchi C, Krahn G, Murray A, Robinson A, Function (2021). State of the Field: The Need for Self-Report Measures of Health and Quality of Life for People With Intellectual and Developmental Disabilities. Journal of Policy and Practice in Intellectual Disabilities.

[CR70] Sikora, D. M., Vora, P., Coury, D. L., Rosenberg, D., & Pediatrics (2012). 130 (Supplement_2),S91-S97. doi:10.1542/peds.2012-0900G 10.1542/peds.2012-0900G 23118259

[CR71] Solberg, B. S., Zayats, T., Posserud, M. B., Halmøy, A., Engeland, A., Haavik, J., & Klungsøyr, K. (2019). Patterns of Psychiatric Comorbidity and Genetic Correlations Provide New Insights Into Differences Between Attention-Deficit/Hyperactivity Disorder and Autism Spectrum Disorder.Biological Psychiatry, 86(8),587–598. doi:10.1016/j.biopsych.2019.04.021 10.1016/j.biopsych.2019.04.021 PMC6764861 31182215

[CR72] StataCorp (2019). Stata Statistical Software: Release 16. In. College Station.

[CR73] Stergiakouli E, Martin J, Hamshere ML, Langley K, Evans DM, Pourcain S, Davey Smith B (2015). Shared Genetic Influences Between Attention-Deficit/Hyperactivity Disorder (ADHD) Traits in Children and Clinical ADHD. Journal of the American Academy of Child & Adolescent Psychiatry.

[CR74] Stern A, Pollak Y, Bonne O, Malik E, Maeir A (2017). The Relationship Between Executive Functions and Quality of Life in Adults With ADHD. Journal Of Attention Disorders.

[CR75] Stice LV, Lavner JA (2019). Social Connectedness and Loneliness Mediate the Association Between Autistic Traits and Internalizing Symptoms Among Young Adults. Journal of Autism and Developmental Disorders.

[CR76] Stickley A, Koyanagi A, Takahashi H, Ruchkin V, Inoue Y, Kamio Y (2017). Attention-deficit/hyperactivity disorder and physical multimorbidity: A population-based study. European Psychiatry.

[CR77] Thomas S, Sciberras E, Lycett K, Papadopoulos N, Rinehart N (2015). Physical Functioning, Emotional, and Behavioral Problems in Children With ADHD and Comorbid ASD: A Cross-Sectional Study. Journal of Attention Disorders.

[CR78] Thorell LB, Holst Y, Sjowall D (2019). Quality of life in older adults with ADHD: links to ADHD symptom levels and executive functioning deficits. Nordic Journal Of Psychiatry.

[CR79] van Heijst BF, Geurts HM (2015). Quality of life in autism across the lifespan: A meta-analysis. Autism.

[CR80] Velilla S (1993). Quantile-based estimation for the Box–Cox transformation in random samples. Statistics & Probability Letters.

[CR81] Vincent A, Da Fonseca D, Baumstarck K, Charvin I, Alcaraz-Mor R, Lehucher-Michel MP (2020). The quality of life and the future of young adults with Asperger syndrome. Disability And Rehabilitation.

[CR82] von Stumm S, Plomin R (2015). Socioeconomic status and the growth of intelligence from infancy through adolescence. Intelligence.

[CR83] WHOQOL Group (1998). Development of the World Health Organization WHOQOL-BREF Quality of Life Assessment. Psychological Medicine.

[CR84] World Health Organization (1996). WHOQOL-BREF: introduction, administration, scoring and generic version of the assessment World Health Organization.

[CR85] World Helath Organization (1995). The World Health Organization quality of life assessment (WHOQOL): Position paper from the World Health Organization. Social Science & Medicine.

[CR86] Yerys, B., McQuaid, G., Lee, N. R., & Wallace, G. (2021). Impacts of Co-Occurring ADHD Symptoms on Critical Markers of Outcome for Autistic Adults. PsyArXiv. doi: 10.31234/osf.io/zwjp7

[CR87] Young S, Adamo N, Ásgeirsdóttir BB, Branney P, Beckett M, Colley W, Woodhouse E (2020). Females with ADHD: An expert consensus statement taking a lifespan approach providing guidance for the identification and treatment of attention-deficit/ hyperactivity disorder in girls and women. Bmc Psychiatry.

